# Depression and anxiety in acute ischemic stroke involving the anterior but not paramedian or inferolateral thalamus

**DOI:** 10.3389/fpsyg.2023.1218526

**Published:** 2023-08-28

**Authors:** Anne-Carina Scharf, Janine Gronewold, Andres Eilers, Olga Todica, Christoph Moenninghoff, Thorsten R. Doeppner, Bianca de Haan, Claudio L. Bassetti, Dirk M. Hermann

**Affiliations:** ^1^Department of Neurology, Institute of Vascular Neurology, Dementia and Ageing Research, University Hospital Essen, University of Duisburg-Essen, Essen, Germany; ^2^Institute of Diagnostic and Interventional Radiology and Neuroradiology, University Hospital Essen, University of Duisburg-Essen, Essen, Germany; ^3^Department of Neurology, University Medical Center Goettingen, Goettingen, Germany; ^4^Division of Psychology, Department of Life Sciences, Centre for Cognitive Neuroscience, Brunel University, London, United Kingdom; ^5^Department of Neurology, University Hospital Bern, Bern, Switzerland

**Keywords:** brain infarct, emotion, depression, anxiety, voxel-based lesion-symptom mapping, magnetic resonance imaging

## Abstract

**Background and objectives:**

Emotional and cognitive deficits are prevalent in strokes involving the thalamus. In contrast to cognitive deficits, emotional deficits have not been studied prospectively in isolated thalamic stroke.

**Methods:**

In 37 ischemic thalamic stroke patients (57.0 [50.0; 69.5] years [median (Q1; Q3)], 21 males, 5 anterior, 12 paramedian, 20 inferolateral vascular territory), and 37 non-stroke control patients matched for age and sex, we prospectively examined depression, anxiety, activities of daily living, and quality of life at 1, 6, 12, and 24 months post-stroke using the Hospital-Anxiety-and-Depression Scale (HADS), Nürnberger-Alters-Alltagsaktivitäten scale (NAA), and Short Form-36 (SF36) questionnaire. Voxel-based lesion-symptom mapping (VLSM) and lesion-subtraction analyzes were performed to determine associations between questionnaire scores and thalamic stroke topography.

**Results:**

At 1 month post-stroke, anterior thalamic stroke patients had higher depression scores [8.0 (7.5; 10.5)] than paramedian [4.5 (1.0; 5.8)] and inferolateral [4.0 (1.0; 7.0)] thalamic stroke patients. Furthermore, anterior thalamic stroke patients had higher anxiety scores [11.0 (8.0; 14.5)] than their matched controls [2.5 (2.0; 2.5)], paramedian [4.5 (1.0; 5.8)] and inferior [4.0 (1.0; 7.0)] thalamic stroke patients. Depression and anxiety scores in anterior thalamic stroke patients remained high across the follow-up [depression: 9.0 (3.5; 13,8); anxiety:10.05 (2.8, 14.5)].

Physical health assessed by SF36 was intact in anterior [1 month post-stroke: T-score = 55.9 (37.0; 57.6)] but reduced in inferolateral [44.5(32.4; 53.1)] thalamic stroke, whereas mental health was reduced in anterior thalamic stroke [32.0 (29.8; 47.3)].

VLSM confirmed that voxels in the anterior thalamus around Montreal Neurological Institute (MNI) coordinates X = −8, Y = −12, Z = 2 were more often affected by the stroke in depressed (HADS-score ≥ 8) than non-depressed (HADS-score < 8) patients and voxels around coordinates X = −10, Y = −12, Z = 2 were more often affected in anxious (HADS-score ≥ 8) than non-anxious (HADS-score < 8) patients.

**Conclusion:**

Anterior, but not paramedian or inferolateral thalamic stroke was associated with depression and anxiety. Even though our results are mostly significant in the left thalamus, this observation on stroke laterality might be confounded by the fact that the right hemisphere was underrepresented in our study.

## Introduction

Depression and anxiety leading to impairments in activities of daily living and quality of life are frequent consequences of ischemic stroke, and are particularly prevalent in patients with stroke lesions involving the thalamus (Hackett and Anderson, 2005; Hermann et al., 2008; Liebermann et al., 2013; Omura et al., 2018). In a retrospective analysis, ⁓70% of thalamic stroke patients had symptoms of depression and anxiety (De Witte et al., 2011). Besides emotional deficits, cognitive deficits were also observed in patients with isolated thalamic stroke, but mostly in single case and retrospective cohort studies (Ghika-Schmid and Bogousslavsky, 2000; Schmahmann, 2003; Carrera and Bogousslavsky, 2006) since isolated thalamic strokes are very rare: Only ⁓1% of all acute ischemic strokes are confined to the thalamus (Schaller-Paule et al., 2021).

Regarding the structural basis of emotional deficits after thalamic stroke, it must be considered that the thalamus represents a complex information processing unit. It is part of multiple neuroanatomical loops, including the medial limbic loop, which connects the anterior thalamus via the cingulate and entorhinal cortex with the hippocampus and mamillary bodies, and a more lateral limbic loop connecting the paramedian thalamus *via* the medial prefrontal and orbitofrontal cortex with the amygdala. For both loops, roles in emotional processing have been described (Papez, 1995; Aggleton et al., 2010; Pessoa, 2017). The different anatomical loops are considered to have an impact on the functional post-stroke outcome in the thalamus.

A stroke in the anterior thalamus, which is part of the medial limbic loop, leads to cognitive impairments in memory, with a focus on anterograde memory loss (Carrera et al., 2004), executive functions, and learning, but also to apathy of the patients (Ghika-Schmid and Bogousslavsky, 2000; Schmahmann, 2003). In a retrospective analysis of 27 paramedian thalamic stroke patients, sleep disturbances and hypersomnia were found 1 month post-stroke (Hermann et al., 2008). In a retrospective cohort study, patients with chronic inferolateral thalamic stroke revealed increased anxiety and reduced quality of life comprising mental and physical health (Liebermann et al., 2013), however with very heterogeneous timing of examinations (37.2 ± 28.4 months post-stroke). In a small cohort of 9 patients with acute inferolateral thalamic stroke, which however spread to the internal capsule in most patients, ⁓45% showed symptoms of longing, apathy, and fear (Prokopiv and Fartushna, 2020).

While we could recently show deficits in verbal memory, language, and executive function in a prospective longitudinal study including 37 acute thalamic stroke patients, cognitive deficits strongly depended on the lesion location within the thalamus (Scharf et al., 2022). Cognitive deficits were most pronounced after infarcts of the anterior thalamus (Scharf et al., 2022). Whether emotional deficits and their long-term recovery also depend on the thalamic lesion site, remained to be elucidated. In his original paper, Papez proposed that the medial limbic loop is involved in emotional processing (Papez, 1995), while this loop has been recognized recently mainly as a circuit for cognitive processes (Aggleton and Brown, 1999; Aggleton et al., 2010). Interestingly, more recent studies suggested a role of the anterior thalamus in emotional processing in patients with treatment-refractory epilepsy, in whom bilateral deep brain stimulation of the anterior thalamus was found to induce reversible depression symptoms (Troster et al., 2017). Furthermore, a single case study of a patient with acute left-sided anterior thalamic infarction reported aspontaneity and loss of psychic self-activation and affective drive, which improved with infarct resorption (Lisovoski et al., 1993).

Based on these previous results and anatomical circuitries of the thalamus, we hypothesized that depression and anxiety are associated with distinct infarct locations within the thalamus, which should be evident in comparison with other infarct locations and with matched controls using methods of lesion-symptom mapping. With the improvement of magnetic resonance imaging (MRI), methods to relate behavioral symptoms to lesion locations such as subtraction analysis and statistical voxel-based lesion-symptom mapping (VLSM) have emerged. In a subtraction analysis, patients are divided into 2 groups based on presence and absence of the symptom of interest. A lesion overlay map is built for both groups, which are then subtracted from each other. This creates a descriptive graphical representation of the brain more frequently damaged in patient with than in patients without the symptom of interest (Rorden and Karnath, 2004). VLSM was developed as a successor of the subtraction analysis and statistically assesses the relation between lesion location and behavioral symptoms (Bates et al., 2003). In the current study, we prospectively examined depression, anxiety, activities of daily living, and health-related quality of life in first-ever ischemic thalamic stroke patients at 1, 6, 12, and 24 months post-stroke in comparison to age- and sex-matched non-stroke control subjects and identified structural lesion correlates using subtraction analysis and VLSM methods.

## Methods

### Study participants

Thirty-seven acute thalamic stroke patients and 37 non-stroke control patients matched for sex and age (≤3 years below or above age of corresponding stroke patient) were prospectively recruited at the University Hospital Essen. Inclusion criteria for stroke patients were an acute isolated first-ever ischemic stroke affecting the thalamus, age ≥ 18 years, no history of neurological or severe psychiatric disorders (e.g., schizophrenia) and no mild psychiatric disorders within the last 5 years (e.g., mild depression). Strokes were identified on 1.5 T MRI scans. We conceptualized a standardized longitudinal test protocol for patients and matched controls. The MRI was carried out specifically for study purposes. It would ethically not have been justifiable to expose the patients and especially the controls to radiation associated with CT scans. Since all MR images for patients and controls were acquired in the same scanner, we reduced potential technical biases. More importantly, we were able to perform brain imaging and neuropsychological examination and questionnaires on the same day. MRI in contrast to CT is known for having a more detailed spatial resolution. Since we delineated our lesions manually, a good resolution was required to outline the stroke borders correctly.

Thalamic strokes were classified into 3 vascular territories as anterior (n = 5), paramedian (*n* = 12) or inferolateral (*n* = 20) thalamic stroke (Bogousslavsky et al., 1988; Morel, 2007). Control patients were age- and sex-matched patients with similar vascular risk profile as stroke patients. They were hospitalized for reasons unrelated to acute or chronic central nervous system pathologies. The study was approved by the Ethics Committee of the University Hospital Essen and all subjects gave written informed consent.

### Demographic and clinical characteristics

Comprehensive neurological and neuropsychological examinations were performed by a neurologist and a neuropsychologist at 1 (mean ± SD 1.2 ± 0.5), 6 (5.7 ± 0.8), 12 (11.8 ± 0.8) and 24 (24.1 ± 1.3) months post-stroke. Demographic and clinical patient characteristics, clinical recovery, depression, anxiety, activities of daily living (ADL), and quality of life were evaluated on occasion of these examinations. Except for the 6 months examination, MRI was carried out on all other examinations at the exact same day as neurological and neuropsychological examination and questionnaires. Data about demographics (age, sex, education), diseases, vascular risk factors, and current medications were collected via detailed structural interview and medical reports. Clinical recovery was assessed by the extended Barthel Index (EBI) (Prosiegel et al., 1996) and the modified Rankin Scale (mRS) (Berger et al., 1999).

### Depression and anxiety evaluated by HADS

Depression and anxiety were assessed by the Hospital Anxiety and Depression Scale (HADS, range 0–21), and scores ≥8 were defined as emotional impairment representing an established cut-off for clinical depression and anxiety (Bjelland et al., 2002).

### ADL evaluated by NAA

ADL were examined using the Nürnberger-Alters-Alltagsaktivitäten scale (NAA, range = 20–60), and scores ≥33 (corresponding to a percentile rank ≤16%) were defined as ADL impairment (Oswald and Fleischmann, 1997).

### Health-related quality of life evaluated by SF36

For the assessment of health-related quality of life, we used the Short Form-36 Health Survey (SF36, T-score for physical and mental health component, range = 27–73) and defined a T-score of ≤40 (corresponding to a percentile rank ≤16%) as quality of life impairment (Ellert and Kurth, 2004).

### MRI data acquisition and image preprocessing

Structural MRI scans were performed on a 1.5 T scanner (MagnetomAvanto, Siemens Healthcare, Erlangen, Germany) with a standard 8-channel birdcage head coil using a 1 mm isotropic 3D T1-weighted volumetric magnetization prepared rapid acquisition gradient echo [MP-RAGE, (TR/TE/TI = 2400/3.52/1200 ms), flip angle = 8°, 256×265 mm^2^ matrix, 1.0×1.0×1.0 mm^3^ resolution, 160 slices] at 1 month post-stroke for the stroke patients and as baseline examination for the control patients.

There is a vivid discussion on the most appropriate interval between stroke onset, neuroimaging, and neuropsychological examinations. An interval between stroke onset and time of image analysis longer than 1 month is not recommended by de Haan and Karnath (2018). The authors claim that post-stroke deficits may recover too quickly, and brain reorganization starts soon after the stroke event. The timing of studies centrally depends on research questions. In our study, the focus was on thalamic areas associated with depression, anxiety, activities of daily living and health-related quality of life. For such studies, de Haan and Karnath recommend using data from an early stroke phase (de Haan and Karnath, 2018). Rennig et al. (2015), however, emphasize that brain swelling in the acute stroke phase impedes image analysis. In order to balance these different arguments, we decided to use MRI data at 1 month post-stroke. At this time-point, all study patients could complete the comprehensive examinations in a stable clinical state, which avoided systematic data biases.

All scans were preprocessed using MRIcroN (Rorden and Brett, 2000) and Statistical Parametric Mapping version 12 (SPM12, https://www.fil.ion.ucl.ac.uk/spm/), running under Matlab R2016a (MathWorks, Natick, MA). Lesion identification was performed by an experienced neuroradiologist. Using MRIcroN, stroke lesion boundaries were manually marked for each patient on the MTI images (Rorden et al., 2007). The lesion map and patient image were subsequently transformed into stereotaxic space using the Clinical Toolbox (Rorden et al., 2012) in SPM12.

The Clinical Toolbox is an age-specific MRI template for normalization. In the spatial normalization process, we fit our patients’ individual MRI scans to this template consisting of MRI scans from patients showing neurological symptoms due to metabolic reasons. The Clinical Toolbox uses SPM’s unified normalization- segmentation approach. For the template creation, an MP-RAGE sequence with 1 mm isotropic voxels, TR = 2,250 ms, TI = 900 ms, TE = 4.52 ms, matrix size = 256 × 256, flip angle = 9°, 160 sagittal slices, was acquired using a Siemens Trio 3 T scanner and 12 element head coil. The elderly clinical population consisted of 30 individuals (17 males) with a mean age of 61.3 years ±18.4 years. The Clinical Toolbox is online available for free.^1^ Cost-function masking was used during the determination of the transformation parameters. Finally, MRIcroN and NPM were used to perform the subtraction and VLSM analyzes. The subtraction analysis is the behavior-based approach in which patients are grouped according to whether or not they show a specific behavioral deficit. The pre-defined scores are used to diagnose the deficit HADS score < 8 (non-impaired) vs. ≥8 (impaired) (Bjelland et al., 2002); NAA scores <33 (non-impaired) vs. ≥33 (impaired); SF36 score > 40 (non-impaired) vs. ≤40 (impaired). We divided our patients into 2 groups based on presence and absence of emotional deficits. A lesion overlay map was built for both groups, which were then subtracted from each other. This results in a graphical representation of the brain areas which are connected to presence of emotional deficits. On the other hand, the VLSM approach eliminates reliance on any cutoff scores, clinical diagnoses, or specified regions of interest. VLSM aims to identify the lesioned brain region with the greatest impact on neuropsychological outcomes and yields valuable insights into the relationship between lesion location and behavior on a voxel-by-voxel basis (Bates et al., 2003).

### Statistical analyzes

Throughout the study, non-parametric tests were applied since Shapiro–Wilk tests showed significant deviations from normal distribution. Demographic and clinical characteristics, depression, anxiety, ADL, and health-related quality of life were compared 1) between thalamic stroke patients and their matched controls and 2) between anterior, paramedian, and inferolateral thalamic stroke patients. Mann–Whitney U tests were used for continuous data (age, education, lesion volume, EBI, mRS, HADS, NAA, SF36) and Fisher’s exact tests for categorical data (sex, vascular risk factors, medications). Results were presented as median (Q1; Q3) for continuous data and as numbers (%) for categorical data. The significance level was set as *p* ≤ 0.05, statistical analyzes were performed with SPSS 22.0 software (SPSS Inc., Chicago, IL, United States).

On the preprocessed MRI scans, we performed VLSM as described by Scharf, Gronewold (Scharf et al., 2022). Lesion-symptom mapping does not require any prior classification of the lesion into groups or locations. It is only important whether a voxel in the MRI image is affected by the stroke or not. In this analysis, we assessed for each voxel, whether voxels status (damaged vs. intact) and emotional status (impaired vs. non-impaired) were significantly associated using the Liebermeister test in NPM (Brunner and Munzel, 2000). Only voxels with a lesion in at least 7 patients were considered to avoid an underpowered analysis as suggested by Moore (2022). To correct for multiple comparisons, a false discovery rate correction was used. Additionally, we performed subtraction analyzes using MRIcroN in which the lesion overlap map from non-impaired patients was subtracted from the lesion overlap map from impaired patients to isolate those areas of the brain more frequently damaged in patients with than in patients without emotional impairment (Rorden and Karnath, 2004). The outcome of the subtraction analyzes is a descriptive graphical representation map of the thalamus region more frequently damaged in patient with than in patients without the symptom of interest.

## Results

### Demographic and clinical characteristics

**Figure 1:**
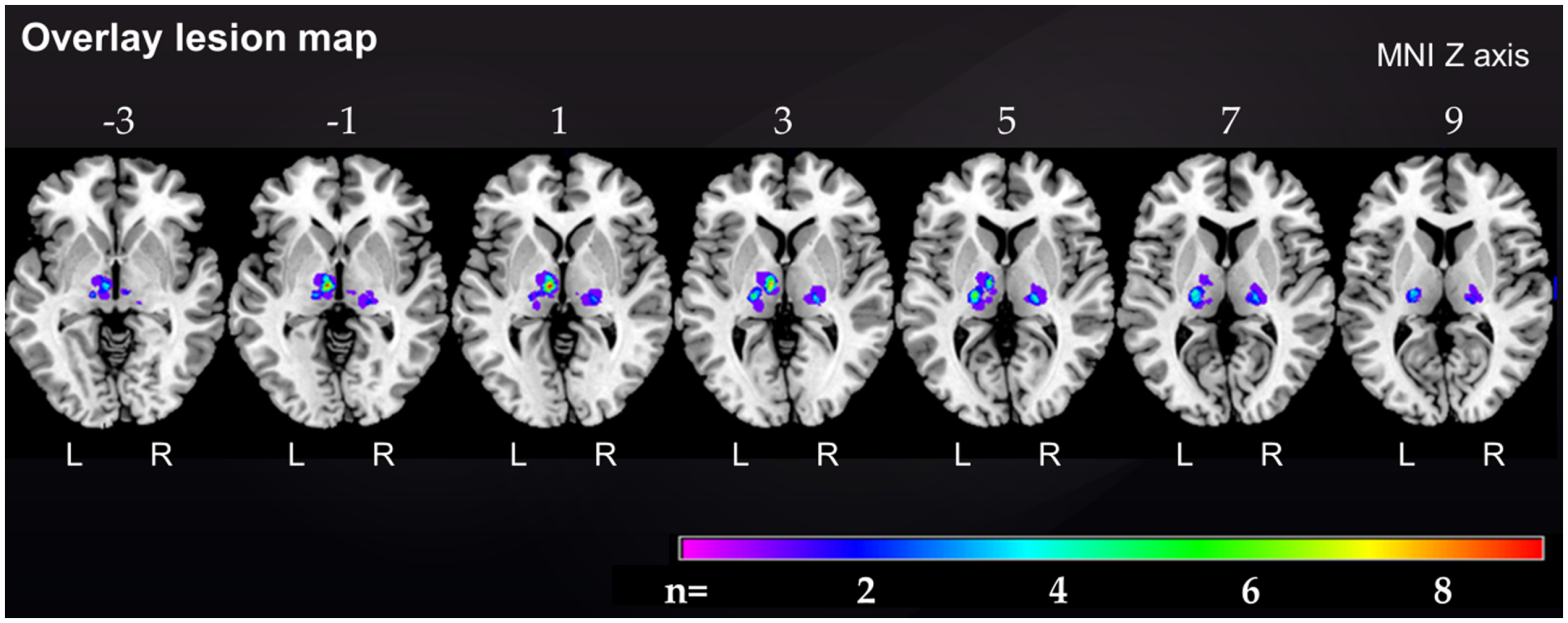
Overlap lesion map of all 37 thalamic stroke patients. The color bar indicates the frequency of lesion overlap in thalamic stroke patients. Numbers above each brain section indicate MNI Z axis. MNI, Montreal Neurological Institute, R, right; L, left.

Demographic and clinical characteristics of stroke patients and their corresponding matched controls are provided in Table 1. The median age of the 37 thalamic stroke patients was 57.0 (50.0, 69.5) years [median (Q1; Q3)], 21 thalamic stroke patients were males, 27 (71.7%) patients had left, 9 (24.3%) had right and 1 (2.6%) had bilateral thalamic stroke. The lesion overlap map of all 37 thalamic strokes (Figure 1) showed that the thalamic regions most often affected were in the left central thalamus (X = −8, Y = −14, Z = 1) with 9 patients showing overlapping lesions. In the left (X = −19, Y = −19, Z = 7) and right (X = 20, Y = −21, Z = 3) inferolateral thalamus, 5 patients each showed overlapping lesions. Inferolateral thalamic stroke patients had significantly more often hyperlipoproteinemia (65.0%) than their matched controls (25.0%, *p* = 0.011). Lesion volume, history of depression, EBI and mRS did not differ between vascular territories affected (Table 1 and Supplementary Table S1). Neurological and cognitive characteristics of the study cohort have been described previously (Scharf et al., 2022).

**Table 1:** Demographic and clinical characteristics of thalamic stroke patients and matched control patients.

	Anterior thalamic stroke patients (*n* = 5)	Paramedian thalamic stroke patients (*n* = 12)	Inferolateral thalamic stroke patients (*n* = 20)	Matched controls anterior thalamic stroke (*n* = 5)	Matched controls paramedian thalamic stroke (*n* = 12)	Matched controls inferolateral thalamic stroke (*n* = 20)
Demographic characteristics						
Age (years)	56.0 (49.0; 58.5)	47.5 (43.3; 65.3)^***^	63.5 (55.3; 73.8)	58.0 (48.5; 72.5)	52.5 (44.3; 66.8)	66.0 (54.3; 72.3)
Sex (male)	4 (80.0)	5 (41.7)	12 (60.0)	4 (80.0)	5 (41.7)	12 (60.0)
Education level (years)	13.0 (9.5; 18.0)	13.0 (10.0; 18.0)^***^	9.0 (8.3; 9.0)^**^	13.0 (10.0; 15.5)	10.0 (10.0; 13.0)	9.5 (9.0; 16.8)
Stroke characteristics						
Lesion side (left)	4 (80.0)	9 (75.0)	14 (66.7)			
Lesion volume in cm^3^	0.49 (0.23; 0.96)	0.20 (0.15; 0.32)	0.25 (0.18; 0.42)			
Vascular risk factors						
Arterial hypertension	3 (60.0)	3 (25.0)^***^	18 (90.0)	3 (60.0)	6 (50.0)	12 (60.0)
Diabetes mellitus	1 (20.0)	1 (8.3)	6 (30.0)	0 (0.0)	1 (8.3)	2 (10.0)
Hyperlipoproteinemia	3 (60.0)	4 (33.3)	13 (65.0)^*^	2 (40.0)	3 (25.0)	5 (25.0)
Obesity (BMI ≥ 25)	3 (60.0)	7 (58.3)	13 (65.0)	4 (80.0)	8 (66.7)	13 (65.0)
Current smoking	3 (60.0)	1 (8.3)	5 (25.0)	1 (20.0)	2 (16.7)	3 (15.0)
History of cardiovascular events in first degree relatives	4 (80.0)	9 (75.0)	14 (70.0)	1 (20.0)	7 (58.3)	14 (70.0)
CNS-affecting drugs						
Anticonvulsants	0 (0.0)	1 (8.3)	0 (0.0)	1 (20.0)	0 (0.0)	2 (10.0)
Tranquilizers	0 (0.0)	1 (8.3)	0 (0.0)	0 (0.0)	1 (8.3)	0 (0.0)
Thyroid hormones	0 (0.0)	3 (25.0)	1 (5.0)	0 (0.0)	1 (8.3)	3 (15.0)
Cortisones	0 (0.0)	0 (0.0)	0 (0.0)	1 (20.0)	0 (0.0)	1 (5.0)
Analgetics	1 (20.0)	1 (8.3)	3 (15.0)	0 (0.0)	0 (0.0)	0 (0.0)

Data are median (Q1; Q3) for continuous data and numbers (%) of patients for categorical data. ^*^p ≤ 0.05 vs corresponding matched controls, ^**^p ≤ 0.05 vs anterior thalamic stroke patients, ^***^p ≤ 0.05 vs inferolateral thalamic stroke patients, ^****^p ≤ 0.05 vs paramedian thalamic stroke patients. CNS, central nervous system.

### Depression and anxiety evaluated by HADS

**Figure 2:**
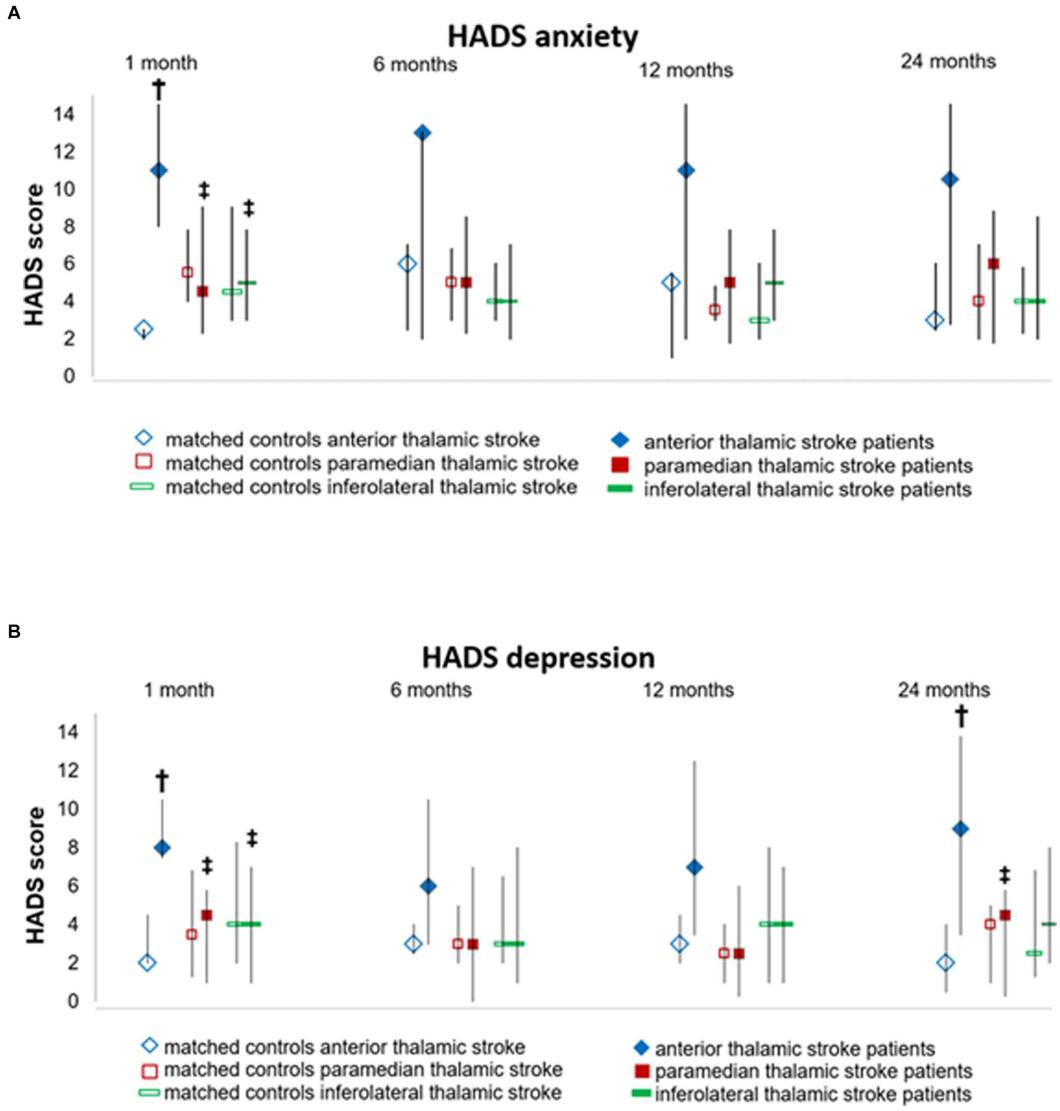
Depression and anxiety assessed by the Hospital Anxiety and Depression Scale (HADS) at different time-points during the follow-up. Data are median (Q1;Q3) of **(A)** depression and **(B)** anxiety scores of thalamic stroke patients and controls., † *p*-value vs. matched controls, ‡ *p*-value vs. anterior thalamic stroke patients.

Anterior thalamic stroke patients had significantly higher HADS depression scores [median (Q1; Q3) = 8.0 (7.5; 10.5)] than paramedian [4.5 (1.0; 5.8), *p* = 0.008] and inferolateral thalamic stroke patients [4.0 (1.0; 7.0), *p* = 0.014] and nominally higher HADS depression scores than their matched controls at 1 month post-stroke [2.0 (2.0; 4.5), *p* = 0.008) (Figure 2 and Supplementary Table S2]. Notably, 4 of the 5 anterior thalamic stroke patients revealed HADS depression scores indicative of clinical depression (Table 2). Depression scores of anterior thalamic stroke patients remained high across the follow-up until 24 months post-stroke. Depression scores in paramedian and inferolateral thalamic stroke patients were low at all time-points examined (Figure 2, Table 2, and Supplementary Table S2).

**Table 2:** Number and percentage of thalamic stroke patients and matched control patients with clinical anxiety and depression, and impairment in activities of daily living and health-related quality of life.

	Anterior thalamic stroke patients (*n* = 5)	Matched controls anterior thalamic stroke (*n* = 5)	Paramedian thalamic stroke patients (*n* = 12)	Matched controls paramedian thalamic stroke (*n* = 12)	Inferolateral thalamic stroke patients (*n* = 20)	Matched controls inferolateral thalamic stroke (*n* = 20)
HADS anxiety (≥8)						
1 month	4 (80.0)	0 (0.0)	3 (25.0)	3 (25.0)	5 (25.0)	7 (35.0)
6 months	3 (60.0)	0 (0.0)	3 (25.0)	1 (8.3)	4 (20.0)	4 (20.0)
12 months	3 (60.0)	0 (0.0)	3 (25.0)	1 (8.3)	5 (25.0)	4 (20.0)
24 months	3 (60.0)	1 (20.0)	4 (33.3)	2 (16.7)	4 (20.0)	3 (15.0)
HADS depression (≥8)						
1 month	4 (80.0)	0 (0.0)	1 (8.3)	2 (16.7)	4 (20.0)	5 (25.0)
6 months	1 (20.0)	0 (0.0)	2 (16.7)	1 (8.3)	6 (30.0)	2 (10.0)
12 months	2 (40.0)	0 (0.0)	0 (0.0)	0 (0.0)	3 (15.0)	5 (25.0)
24 months	3 (60.0)	0 (0.0)	0 (0.0)	1 (8.3)	3 (15.0)	3 (15.0)
NAA (≥33)						
1 month	0 (0.0)	0 (0.0)	3 (25.0)	1 (8.3)	4 (20.0)	1 (5.0)
6 months	2 (40.0)	0 (0.0)	2 (16.7)	0 (0.0)	3 (15.0)	1 (5.0)
12 months	1 (20.0)	0 (0.0)	1 (8.3)	1 (8.3)	3 (15.0)	0 (0.0)
24 months	2 (40.0)	0 (0.0)	1 (8.3)	1 (8.3)	2 (10.0)	0 (0.0)
SF36 physical health (≤40)						
1 month	1 (20.0)	2 (40.0)	3 (25.0)	5 (41.7)	9 (45.0)	4 (20.0)
6 months	2 (40.0)	2 (40.0)	2 (16.7)	4 (33.3)	8 (40.0)	5 (25.0)
12 months	0 (0.0)	1 (20.0)	2 (16.7)	3 (25.0)	10 (50.0)	3 (15.0)
24 months	1 (20.0)	2 (40.0)	3 (25.0)	3 (25.0)	7 (35.0)	4 (20.0)
SF36 mental health (≤40)						
1 month	4 (80.0)	0 (0.0)	2 (16.7)	2 (16.7)	4 (20.0)	7 (35.0)
6 months	2 (40.0)	0 (0.0)	4 (33.3)	1 (8.3)	2 (10.0)	4 (20.0)
12 months	2 (40.0)	0 (0.0)	3 (25.0)	0 (0.0)	4 (20.0)	2 (10.0)
24 months	2 (40.0)	0 (0.0)	3 (25.0)	1 (8.3)	2 (10.0)	4 (20.0)

HADS, Hospital Anxiety and Depression Scale; NAA, Nürrnberger-Alters-Alltagsaktivitäten scale; SF36, Short Form-36.

Anterior thalamic stroke patients had significantly higher HADS anxiety scores [11.0 (8.0; 14.5)] than their matched controls (2.5 (2.0; 2.5), *p* = 0.012), paramedian thalamic stroke patients [4.5 (2.3; 9.0), *p* = 0.030], and inferolateral thalamic stroke patients [5.0 (2.5; 7.8), *p* = 0.009] at 1 month post-stroke (Figure 2 and Supplementary Table S2). Four of the 5 anterior thalamic stroke patients revealed a HADS anxiety score indicative of clinical anxiety. Anxiety scores in anterior thalamic stroke patients remained high across the follow-up. Anxiety scores in paramedian and inferolateral thalamic stroke patients were low at all time-points (Figure 2, Table 2, and Supplementary Table S2).

### ADL evaluated by NAA

Thalamic stroke patients did not exhibit meaningful ADL impairment. Only anterior thalamic stroke patients [30.0 (28.5; 31.5)] reported significantly higher NAA scores (indicative of lower ADL) than their corresponding matched controls [25.0 (23.3; 27.5), *p* = 0.019] at 1 month post-stroke (Supplementary Table S2). NAA scores in anterior thalamic stroke patients remained high across the follow-up (Table 2 and Supplementary Table S2).

### Health-related quality of life evaluated by SF36

Physical health was well preserved in anterior thalamic stroke patients. Their physical health scores [55.9 (37.0; 57.6)] were nominally higher (indicating better health) than in paramedian [45.9 (37.7; 55.5)] and inferolateral thalamic stroke patients [44.5 (32.4; 53.1)] at 1 month post-stroke, which persisted at all follow-ups (Supplementary Table S2). Physical health in inferolateral thalamic stroke patients was significantly lower than in their corresponding matched controls [53.6 (41.3; 57.1), *p* = 0.037] at 1 month post-stroke (Supplementary Table S2) and remained low at 24 months post-stroke ([36.0 (21.2; 52.5)], Table 2, and Supplementary Table S2).

Mental health in anterior thalamic stroke patients [32.0 (29.8; 47.3)] was significantly lower (indicating poorer health) than in their matched controls [55.2 (49.4; 57.5), *p* = 0.047] and inferolateral thalamic stroke patients [49.9 (40.9; 56.3), *p* = 0.030] at 1 month post-stroke (Supplementary Table S2). Mental health scores in anterior thalamic stroke patients remained low across the follow-up [24 months: 37.9 (24.5; 51.9], Table 2, and Supplementary Table S2).

### VLSM and lesion-subtraction analysis for depression and anxiety, ADL, and health-related quality of life of thalamic stroke patients

#### VLSM for HADS depression and anxiety

**Figure 3:**
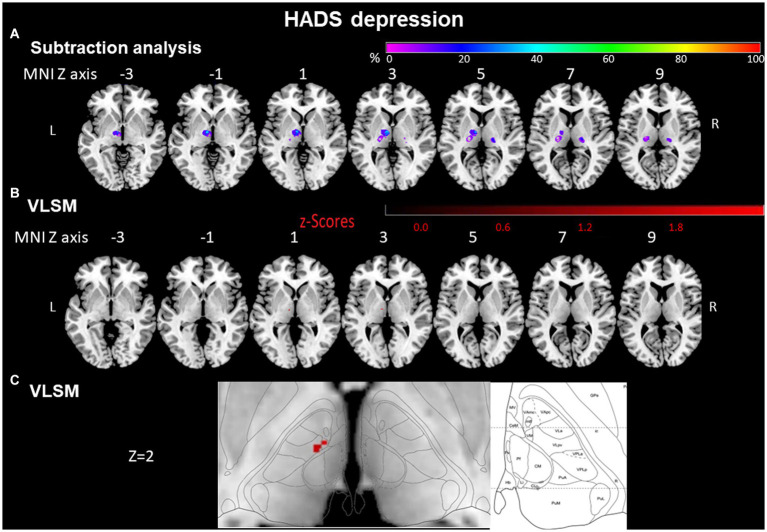
HADS depression evaluated by subtraction analysis and voxel-based symptom-lesion mapping (VLSM). **(A)** Subtraction analysis in which a lesion overlay of non-depressed patients (HADS score < 8) was subtracted from a lesion overlay of depressed patients (HADS score ≥ 8) demonstrating the lesion topography of depressed patients. The color bar indicates a relative differences value by how much more frequently (in percentage) each voxel was damaged in the depressed patient group than in non-depressed patient group (from purple = 0% = voxel was damaged equally in both groups to red = 100% = voxel was damaged in every patient in the depressed patient group but in none of the non-depressed patient group). **(B)** VLSM analysis for depressed (HADS score ≥ 8) vs. non-depressed (HADS score < 8) patients. The color bar demonstrates z-values of the Liebermeister tests. **(C)** Enlargement of **(B)** at Z coordinate 2 next to the lesion location mapping according to Morel’s anatomical thalamic atlas. Numbers above each brain indicate MNI Z coordinates. Only voxels significant at *p* ≤ 0.05 are shown. MNI, Montreal Neurological Institute, R, right; L, left.

VLSM confirmed that injury to the anterior thalamus was associated with depression and anxiety. Voxels around Montreal Neurological Institute (MNI) coordinates X = -8, Y = -12, Z = 2 in the left anterior thalamus were significantly more often affected by the stroke in depressed than in non-depressed patients (Liebermeister test, *p* = 0.020). According to Morel’s anatomical thalamic atlas (Morel, 2007), these lesions cover the ventral lateral and ventral medial thalamic nuclei (Figures 3B,C).

**Figure 4:**
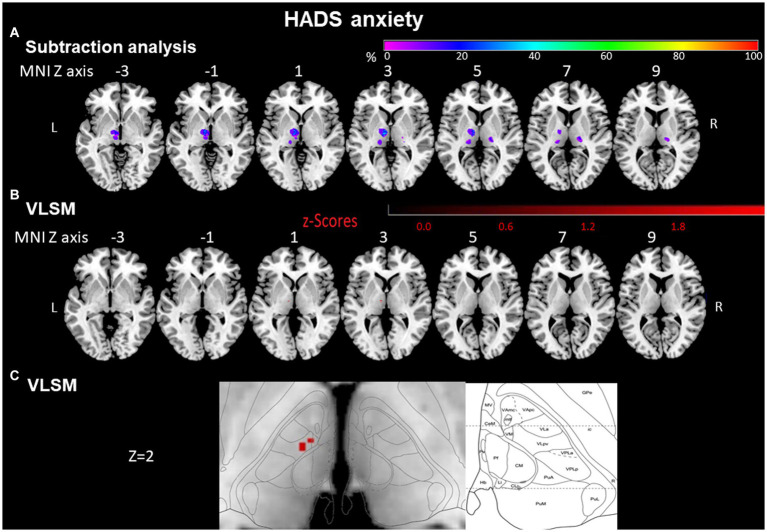
HADS anxiety assessed by subtraction analysis and VLSM. **(A)** Subtraction analysis in which a lesion overlay of non-anxious patients (HADS score < 8) was subtracted from a lesion overlay of anxious patients (HADS score ≥ 8) demonstrating the lesion topography of anxious patients. The color bar indicates a relative differences value by how much more frequently (in percentage) each voxel was damaged in the anxious patient group than in non-anxious patient group (from purple = 0% = voxel was damaged equally in both groups to red = 100% = voxel was damaged in every patient in the anxious patient group but in none of the non-anxious patient group). **(B)** VLSM analysis for anxious (HADS score ≥ 8) vs. non-anxious (HADS score < 8) patients. The color bar demonstrates z-values of the Liebermeister tests. **(C)** Enlargement of **(B)** at Z coordinate 2 next to the lesion location mapping according to Morel’s anatomical thalamic atlas. Numbers above each brain indicate MNI Z coordinates. Only voxels significant at *p* ≤ 0.05 are shown. MNI, Montreal Neurological Institute, R, right; L, left.

Voxels around MNI coordinates X = −10, Y = −12, Z = 2 in the left anterior thalamus were significantly more often affected by stroke in anxious than in non-anxious patients (Liebermeister test, *p* = 0.003, FDR corrected <0.05). These MNI coordinates cover the ventral lateral and ventral medial nuclei of the thalamus (Figures 4B,C).

To validate our results, we performed a statistical comparison between patients with a lesion in the specific voxels identified by VLSM and without that lesion. These analyzes showed significant differences a) for HADS anxiety (U = 41.5, *p* = 0.009) and b) for HADS depression (U = 33.5, *p* = 0.003).

#### Subtraction analysis for HADS depression

In a lesion subtraction analysis, in which a lesion overlay of non-depressed patients was subtracted from a lesion overlay of depressed patients, the left anterior thalamic nuclei (X = −9, Y = −10, Z = 0) were damaged at least 50% more frequently in the depressed than the non-depressed group (Figure 3A).

The voxels around the area which were significantly affected in the VLSM (X = −8, Y = −12, Z = 2) were also covered by the subtraction analysis and were damaged at least 50% more frequently in the depressed than the non-depressed group.

#### Subtraction analysis for HADS anxiety

In a lesion-subtraction analysis, in which a lesion overlay of non-anxious patients was subtracted from a lesion overlay of anxious patients, the left anterior thalamic nuclei (X = −9, Y = −10, Z = 3) were damaged at least 50% more frequently in the anxious than the non-anxious group (Figure 4A). The voxels around the area which were significantly affected in the VLSM (X = −10; Y = −12; Z = 2) were also covered by the subtraction analysis and were damaged at least 50% more frequently in the anxious than the non- anxious group.

*VLSM for NAA and SF36.* In VLSM, impairment in ADL and physical and mental health were not associated with specific voxel damage (NAA Liebermeister, *p* = 0.189; SF36 physical health Liebermeister test, *p* = 0.509; SF36 mental health Liebermeister test, *p* = 0.500).

#### Subtraction analysis for NAA and SF36

**Figure 5:**
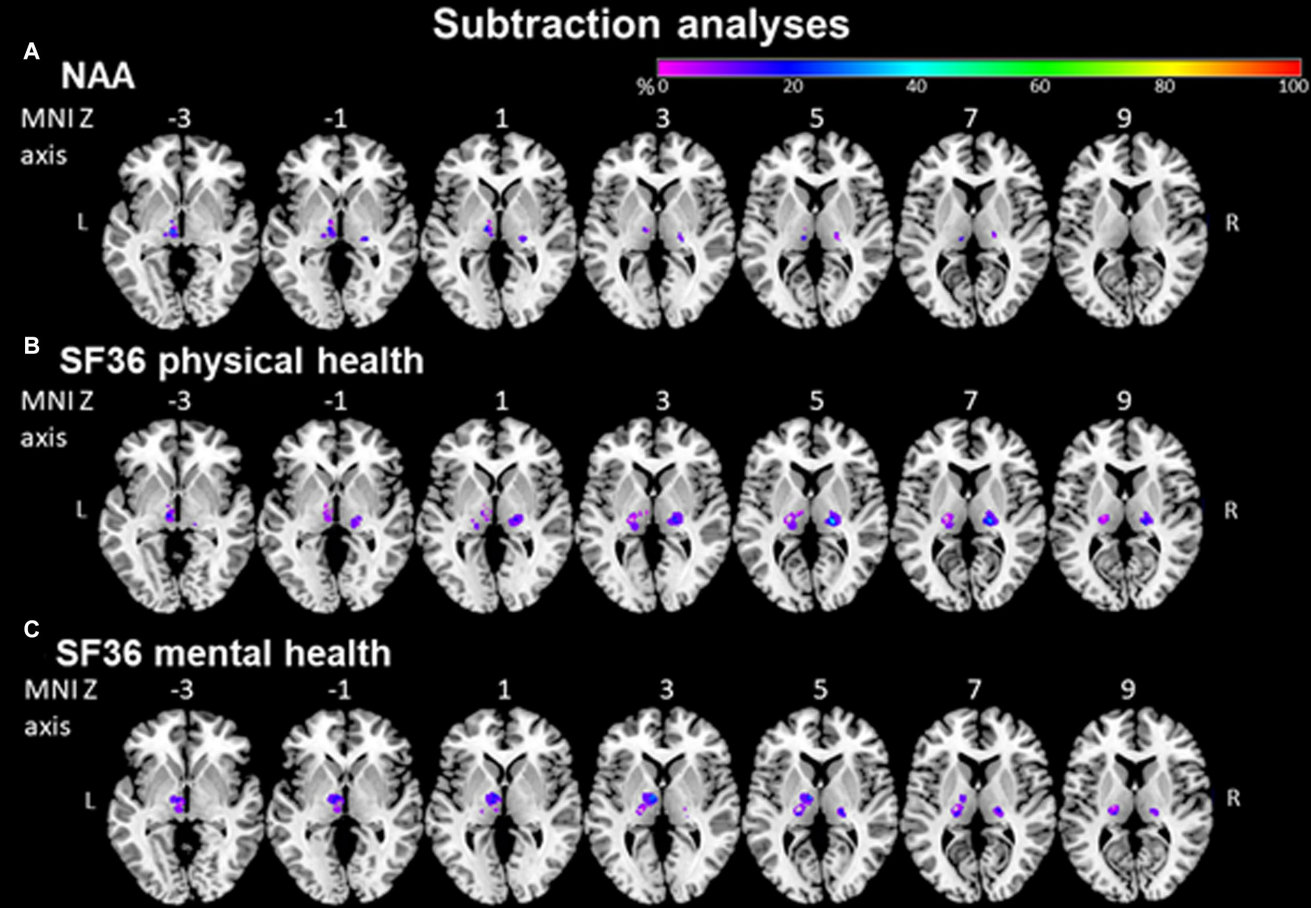
NAA and SF36 evaluated by subtraction analysis. **(A)** Subtraction analysis in which a lesion overlay of patients without ADL impairment (NAA score < 33) was subtracted from a lesion overlay of patients with ADL impairment (NAA score ≥ 33) demonstrating the lesion topography of ADL impaired patients. The color bar indicates a relative differences value by how much more frequently (in percentage) each voxel was damaged in ADL impaired than in ADL non-impaired patients (from purple = 0% = voxel was damaged equally in both groups to red = 100% = voxel was damaged in every patient in the ADL impaired patient group but in none of the ADL non-impaired patient group). **(B)** + **(C)** Subtraction analysis where a lesion overlay of patients without physical/mental health impairment (SF36 > 40) was subtracted from a lesion overlay of patients with physical/mental health impairment (SF36 ≤ 40) demonstrating the lesion topography of physical/mental health-impaired patients. The color bar indicates a relative differences value by how much more frequently (in percentage) each voxel was damaged in physical/mental health impaired than in physical/mental health non-impaired patients (from purple = 0% = voxel was damaged equally in both groups to red = 100% = voxel was damaged in every patient in the physical/mental health impaired patient group but in none of the physical/mental health non-impaired patient group).

In a lesion-subtraction analysis, in which a lesion overlay of patients without ADL impairment was subtracted from a lesion overlay of patients with ADL impairment, the left inferolateral thalamus (X = −10, Y = −16, Z = 0) was damaged at least 40% more frequently in patients with than in patients without ADL impairment (Figure 5A).

In a lesion-subtraction analysis, in which a lesion overlay of patients without physical health impairment was subtracted from a lesion overlay of patients with physical health impairment, the right inferolateral thalamus (X = −21, Y = 19, Z = 5) was damaged at least 40% more frequently in patients with than in patients without physical health impairment (Figure 5B).

In a lesion-subtraction analysis, in which a lesion overlay of patients without mental health impairment was subtracted from a lesion overlay of patients with mental health impairment, the left anterior thalamus (X = −8, Y = −9, Z = 4) was damaged at least 35% more frequently in patients with than in patients without mental health impairment (Figure 5C). Since VLSM could not show any significant voxels for the NAA and SF36 questionnaires, we performed this comparison using the voxels identified by subtraction analyzes. There was no significant difference between patients with stroke in the voxels identified by subtraction analyzes and without stroke in these voxels concerning ADL impairment (NAA; U = 89.0, *p* = 0.376) and physical (U = 40.0, *p* = 0.112) and mental (U = 41.0, *p* = 0.135) health impairment (SF36).

VLSM is a tool established based on concepts of subtraction analysis. The purpose of subtraction analysis is mainly to provide graphical information about the connection between damaged thalamic brain areas and emotional deficits, while VLSM allows detailed statistical significance analysis. For statistical comparisons, VLSM is preferable to subtraction analysis.

## Discussion

In this prospective longitudinal case–control study, we observed that isolated ischemic stroke of the anterior, but not paramedian or inferolateral thalamus, was associated with elevated levels of depression and anxiety assessed by HADS. Patients with anterior thalamic stroke did not report major deficits in physical health evaluated by SF36 but did show lower levels of mental health, that persisted over 24 months. VLSM showed that voxels around MNI coordinates X = −8, Y = −12, Z = 2 in the left anterior thalamus were significantly more often affected by stroke in depressed than in non-depressed patients. Voxels around MNI coordinates X = −10, Y = −12, Z = 2 in the left anterior thalamus were significantly more often affected by stroke in anxious than in non-anxious patients. These finding suggest a role of the anterior thalamus in emotional processing.

### Results in context of existing literature

Previous cohort studies suggested that depression is particularly prevalent in patients with stroke lesions involving the thalamus (Hackett and Anderson, 2005; Omura et al., 2018). In a retrospective analysis of 75 thalamic stroke patients, DeWitte et al. observed depression and anxiety symptoms in ⁓70% of all patients, independent of the vascular territory affected (De Witte et al., 2011). Previous studies suggested a prevalence of 20–55% for symptoms of depression and of ⁓20% for symptoms of anxiety for strokes not confined to the thalamus (Campbell Burton et al., 2013). This could imply that depression and anxiety are indeed more frequent in thalamic than non-thalamic stroke patients. In our study, HADS depression and anxiety scores were elevated in patients with anterior thalamic stroke. Hence, 4 of the 5 anterior thalamic stroke patients exhibited depression defined by HADS scores ≥8. Conversely, depression and anxiety scores were not increased in paramedian and inferolateral thalamic strokes in comparison to control subjects without stroke. While post-stroke depression in general persists for 3 to 6 months post-stroke (Barker-Collo, 2007), anterior thalamic stroke patients in this study still exhibited elevated depression scores after 2 years. Only anterior thalamic stroke patients but not paramedian and inferolateral thalamic stroke patients reported significantly higher NAA scores (indicative of lower ADL) when compared to their corresponding matched controls. As a matter of fact, depression may exacerbate behavioral deficits and ADL impairments which in turn may further aggravate mood disturbances in anterior thalamic stroke (Ghaffari et al., 2020). Our study did not discriminate whether post-stroke depression was a direct consequence of brain injury or a functional response of stroke-associated neurological deficits. Future studies should focus on this topic.

In an earlier study, Liebermann et al. (2013) compared the outcome of 68 chronic thalamic stroke patients at 37.2 ± 28.4 months post-stroke with 34 transient ischemic attack patients. In anterior thalamic stroke patients, no depression or anxiety and a normal health-related quality of life were noted. In this study, HADS anxiety and SF12 physical and mental health scores, but not HADS depression scores were increased in posterior thalamic stroke patients, which according to the protocol of that study comprised paramedian and inferolateral thalamic stroke patients (Liebermann et al., 2013). Inferolateral thalamic strokes frequently induce sensorimotor deficits with daily life impairments. The different timing of patient evaluation, which in the case of the previous study was >3 years post-stroke, may explain the different findings of the present and this earlier study influenced by post-stroke reorganization of the brain. Supporting this hypothesis of post-stroke reorganization of the brain, a study including 24 cases of thalamic infarction or hemorrhage involving the anterior thalamic nucleus or mammillothalamic tract observed ipsilateral atrophy of the mammillary body in almost half of the patients and ipsilateral atrophy of the fornix and almost 10% of the patients within a mean follow-up of 2.5 ± 1.4 years (Kinoshita et al., 2019). In line with our study results, several case studies suggest a role of the anterior thalamus in emotional processing. An analysis of 3 personal and 5 published cases with acute infarction in the anterior thalamus (6 left-sided, 3 right-sided) revealed symptoms of apathy (Bogousslavsky et al., 1986). A study of a single case with acute right-sided infarction of the anterior thalamus observed new onset aggressive behavior, which could successfully be treated with divalproex sodium, citalopram and olanzapine (Srivastava et al., 2013). Contrary to our results, in a small cohort of 9 patients with acute inferolateral thalamic stroke, which however spread to the internal capsule in most patients, ⁓45% showed symptoms of longing, apathy, and fear. Patients with anterior thalamic stroke were not included in this earlier study (Prokopiv and Fartushna, 2020). Based on our and previous studies together with anatomical evidence, it can be concluded that the anterior thalamic nuclei play a role in emotion processing, most likely by connecting the anterior thalamus to the limbic system via the cingulate cortex and entorhinal cortex, the so-called medial limbic loop (Schmahmann, 2003; Chen et al., 2016). Papez (1995) already proposed that the medial limbic loop is involved in emotional processing, but the role of this loop in emotional processing may have been underestimated so far.

### Clinical implications

Our study results have important clinical implications. Post-stroke impairments in emotional processing such as depression and anxiety are associated with poorer recovery affecting physical and cognitive function, quality of life and even mortality (Hermann et al., 2008; Goodin et al., 2019). Thus, it is important to identify and treat those “at risk” of developing post-stroke impairments in emotional processing early to improve recovery. Despite evidence still being scarce, single case studies already reported successful treatment of impairments in emotional processing following thalamic stroke by use of antidepressants, antiepileptics, and stimulants (Srivastava et al., 2013; Aragona et al., 2018).

### Strengths and limitations

To the best of our knowledge, this is the first longitudinal case–control study, which prospectively investigated impairments in emotion, ADL, and quality of life in acute ischemic thalamic stroke patients. All other studies published so far are either case studies or, if they have a larger sample size, they are based on chronic thalamic infarcts with different intervals between stroke onset and examination.

The major strength of our study is the combination of two evaluation methods. In the first method, we compare the outcome of our questionnaires regarding depression, anxiety, activities of daily living, and quality of life between our anterior, paramedian, and inferolateral thalamic stroke patients based on vascular territory affected. In the VLSM method, we renounced this group classification to reevaluate effects of lesion locations without prior assumptions. When performing between group comparisons, the different patients’ characteristics age and educational levels could bias the statistical analysis with the well-known inferential statistical methods. For this reason, we applied additional analysis methods to our data with subtraction analysis and VLSM, which analyze the data as a collective without prior assumptions. The two evaluation methods provide converging evidence suggesting that damage to areas in the anterior thalamus in particular are associated with depression and anxiety.

Due to the prospective design, the numbers of stroke patients were not equally distributed and not matched within different thalamic stroke topography groups in our study. Most strokes (54.1%) affected the inferolateral thalamus and 13.5% the anterior thalamus. This distribution may limit the extrapolation of our data, but is comparable to Liebermann et al. (2013) and Carrera and Bogousslavsky (2006) who described that 8.8 and 13%, respectively, of their study cohort had anterior thalamic stroke.

The stroke topography groups had different vascular risk profiles, although they differ not significantly. Studies, focusing on the connection between smoking and depression or anxiety, showed that there is a close connection between smoking and depression or anxiety. In anterior thalamic stroke patients, smoking was prevalent in 60% of the patient, which may influence the high scores in depression and anxiety (Byeon, 2015; Alizadeh et al., 2021). A population-based study could demonstrate the influence of arterial hypertension on depression, which is prevalent in 90% of our inferolateral thalamic stroke patients (Villarreal-Zegarra and Bernabe-Ortiz, 2020). In conceptualizing the study, we were aware of the impact of vascular risk factors on mood and quality of life. Based on this, we decided that the control group should not consist of healthy subjects, but also have vascular risk factors and comorbidities. These risk factors such as smoking and arterial hypertension also contribute to the occurrence of depression and anxiety in the control group. The anterior thalamic stroke patients are healthier than the patients of the other stroke (e.g., less medication) locations and this is also reflected in their matched controls. Therefore, these matched controls also have a lower risk of depression and anxiety. There was no statistical difference between the matched control groups.

Since more patients had a left-sided than a right-sided stroke, symptom-lesion mapping might be biased towards the left hemisphere. Future studies should evaluate more systematically the prevalence of depression and anxiety in right-sided thalamic stroke patients. Our observations might also be influenced by differences of the lesion volume, which was nominally higher in anterior than in paramedian or inferolateral stroke. Associations between lesion volume and levels of depression and anxiety have previously been demonstrated for other stroke locations (Nickel and Thomalla, 2017). Yet, in our study the lesion volume was not significantly correlated to depression (*r* = 0.217, *p* = 0.225) and anxiety (*r* = 0.143, *p* = 0.428) in the HADS.

Given that acute thalamic strokes are rare events (Nolte et al., 2011), the number of 37 stroke patients included in our study is considerable. We asked the patients about their psychiatric history and medications and also checked their medical records. However, we did not perform psychological testing to analyze pre-stroke depression and anxiety levels. Hence, we cannot completely rule out that the higher levels of depression and anxiety in the anterior thalamic stroke patients were not already present at baseline, especially when considering the small sample size of anterior thalamic stroke patients.

Finally, we have to acknowledge that the HADS is a screening tool which does not evaluate diagnostic DSM-V or ICD-10 depression or anxiety criteria (Aben et al., 2002). The advantage of the HADS is that it provides quantitative scores, suitable for statistical analysis, which identify depression and anxiety in an efficient, reliable and valid way (Bjelland et al., 2002). In the paramedian group two of the patients used an antidepressant as concomitant medication, which could have a mood enhancing effect on them.

## Summary/conclusions

This study demonstrates that anterior but not inferolateral or paramedian thalamic stroke is associated with depression and anxiety. VLSM identified voxels in the anterior thalamus that were preferentially affected by the stroke in patients with depression and anxiety.

## Data availability statement

The original contributions presented in the study are included in the article/Supplementary material, further inquiries can be directed to the corresponding author.

## Ethics statement

The studies involving humans were approved by ethics committee of the university Hospital Essen. The studies were conducted in accordance with the local legislation and institutional requirements. The participants provided their written informed consent to participate in this study.

## Author contributions

A-CS, JG, AE, OT, CM, TD, BH, and DH substantial contributions to the conception or design of the work, or the acquisition, analysis, or interpretation of data for the work. A-CS, JG, TD, BH, CB, and DH drafting the work or revising it critically for important intellectual content. A-CS, JG, AE, OT, CM, TD, BH, CB, and DH provide approval for publication of the content. A-CS, JG, CM, TD, BH, CB, and DH agree to be accountable for all aspects of the work in ensuring that questions related to the accuracy or integrity of any part of the work are appropriately investigated and resolved. All authors contributed to the article and approved the submitted version.

## Conflict of interest

The authors declare that the research was conducted in the absence of any commercial or financial relationships that could be construed as a potential conflict of interest.

## Publisher’s note

All claims expressed in this article are solely those of the authors and do not necessarily represent those of their affiliated organizations, or those of the publisher, the editors and the reviewers. Any product that may be evaluated in this article, or claim that may be made by its manufacturer, is not guaranteed or endorsed by the publisher.

## Footnotes

^1^ https://www.nitrc.org/projects/clinicaltbx/
