# Supplementary material for: Depression and anxiety in acute ischemic stroke involving the anterior but not paramedian or inferolateral thalamus
Source: Front Psychol. 2023 Aug 28;14:1218526. doi: 10.3389/fpsyg.2023.1218526 (PMC10493383; doi:10.3389/fpsyg.2023.1218526)
Supplement: Supplementary file 1 [file Data_Sheet_1.PDF]

Depression and anxiety in acute ischemic stroke involving the anterior but not paramedian or inferolateral thalamus

Authors:
Anne-Carina Scharf, MD, MSc,[1] Janine Gronewold, PhD,[1] Andres Eilers, MD, [1] Olga Todica, MSc,[1] Christoph Moenninghoff, MD,[2] Thorsten R. Doeppner, MD, MSc,[3] Bianca de Haan, PhD,[4] Claudio L. Bassetti, MD,[5] Dirk M. Hermann, MD[1]

Institutions:
[1]Department of Neurology and [2]Institute of Diagnostic and Interventional Radiology and Neuroradiology, University Hospital Essen, University of Duisburg-Essen, Essen, Germany; [3]Department of Neurology, University Medical Center Goettingen, Goettingen, Germany; [4]Division of Psychology, Department of Life Sciences, Centre for Cognitive Neuroscience, Brunel University, London, U.K.; [5]Department of Neurology, University Hospital Bern, Bern, Switzerland

Correspondence:

Name:	Prof. Dirk M. Hermann, MD
Mailing address:	Chair of Vascular Neurology, Dementia and Ageing Research<br>Department of Neurology, Hufelandstraße 55, 45147 Essen, Germany
Telephone:	+ 49 201 723- 2180
Fax number:	+ 49 201 723- 5534
Email:	dirk.hermann@uk-essen.de

<u>Table of content</u>

Table S1. Extended Barthel Index (EBI) and modified Rankin Scale (mRS) in thalamic stroke patients and matched control patients

Table S2. Depression and anxiety, activities of daily living and health-related quality of life of thalamic stroke patients and matched control patients

Figure S1. Thalamic stroke patient recruitment. Patients included and excluded at various points of examination are shown.

Table S1 Extended Barthel Index (EBI) and modified Rankin Scale (mRS) in thalamic stroke patients and matched control patients

	Anterior thalamic stroke patients (n=5)	Paramedian thalamic stroke patients (n=12)	Inferolateral thalamic stroke patients (n=20)	Matched controls anterior thalamic stroke (n=5)	Matched controls paramedian thalamic stroke (n=12)	Matched controls inferolateral thalamic stroke (n=20)
Extended Barthel Index						
Pre-stroke	190.0 (187.5;190.0)	190.0 (190.0;190.0)	190.0 (190.0;190.0)	190.0 (190.0;190.0)	190.0 (190.0;190.0)	190.0 (190.0;190.0)
1 month	185.0 (167.5;190.0)	190.0 (186.3;190.0)	190.0 (181.3;190.0)	190.0 (190.0;190.0)	190.0 (190.0;190.0)	190.0 (190.0;190.0)
6 months	185.0 (175.0;187.5)	190.0 (190.0;190.0)	190.0 (188.8;190.0)	190.0 (190.0;190.0)	190.0 (190.0;190.0)	190.0 (190.0;190.0)
12 months	185.0 (175.0;187.5)	190.0 (190.0;190.0)	190.0 (190.0;190.0)	190.0 (190.0;190.0)	190.0 (190.0;190.0)	190.0 (190.0;190.0)
24 months	185.0 (177.5;188.8)	190.0 (190.0;190.0)	190.0 (187.5;190.0)	190.0 (190.0;190.0)	190.0 (190.0;190.0)	190.0 (190.0;190.0)
Modified Rankin Scale						
1 month	2.0 (1.5;2.0)	2.0 (1.3;2.0)	2.0 (1.0;2.0)			
6 months	1.0 (0.0;2.0)	1.0 (0.0;1.0)	1.0 (1.0;2.0)			
12 months	1.0 (0.5;2.0)	1.0 (0.0;1.0)	1.0 (1.0;2.0)			
24 months	1.5 (0.3;2.0)	0.5 (0.0;1.0)	1.5 (1.0;2.3)			

Data are median (Q1;Q3)

Table S2 Depression and anxiety, activities of daily living and health-related quality of life of thalamic stroke patients and matched control patients

	Anterior thalamic stroke patients (n=5)	Paramedian thalamic stroke patients (n=12)	Inferolateral thalamic stroke patients (n=20)	Matched controls anterior thalamic stroke (n=5)	Matched controls paramedian thalamic stroke (n=12)	Matched controls inferolateral thalamic stroke (n=20)
HADS anxiety						
1 month	11.0 (8.0;14.5)†	4.5 (2.3;9.0)‡	5.0 (3.0;7.8)‡	2.5 (2.0;2.5)	5.5 (4.0;7.8)	4.5 (3.0;9.0)
6 months	13.0 (2.0;13.0)	5.0 (2.3;8.5)	4.0 (2.0;7.0)	6.0 (2.5;7.0)	5.0 (3.0;6.8)	4.0 (3.0;6.0)
12 months	11.0 (2.0;14.5)	5.0 (1.8;7.8)	5.0 (3.0;7.8)	5.0 (1.0;5.5)	3.5 (3.0;4.8)	3.0 (2.0;6.0)
24 months	10.5 (2.8;14.5)	6.0 (1.8;8.8)	4.0 (2.0;8.5)	3.0 (2.5;6.0)	4.0 (2.0;7.0)	4.0 (2.3;5.8)
HADS depression						
1 month	8.0 (7.5;10.5)†	4.5 (1.0;5.8)‡	4.0 (1.0;7.0)‡	2.0 (2.0;4.5)	3.5 (1.3;6.8)	4.0 (2.0;8.3)
6 months	6.0 (3.0;10.5)	3.0 (0.0;7.0)	3.0 (1.0;8.0)	3.0 (2.5:4.0)	3.0 (2.0;5.0)	3.0 (2.0;6.5)
12 months	7.0 (3.5;12.5)	2.5 (0.3;6.0)	4.0 (1.0;7.0)	3.0 (2.0;4.5)	2.5 (1.0;4.0)	4.0 (1.0;8.0)
24 months	9.0 (3.5;13.8)†	4.5 (0.3;5.8)‡	4.0 (2.0;8.0)	2.0 (0.5;4.0)	4.0 (1.0;5.0)	2.5 (1.3;6.8)
NAA						
1 month	30.0 (28.5;31.5)†	28.5 (23.0;32.8)	27.5 (24.3;31.8)	25.0 (23.3;27.5)	26.5 (23.3;29.5)	25.0 (23.0;27.8)
6 months	26.0 (23.5;40.5)	25.5 (22.0;28.8)	26.0 (24.0;30.0)	26.0 (24.5;27.5)	24.5 (22.3;26.0)	26.5 (23.0;28.0)
12 months	28.0 (25.0;37.5)	26.0 (23.0;30.5)	28.0 (26.0;32.0)†	26.0 (24.0;27.0)	24.0 (22.5;26.5)	26.0 (22.3;27.8)
24 months	31.0 (23.5;39.3)	26.0 (22.0;30.0)	27.0 (24.5;30.0)	24.0 (21.5;29.5)	25.0 (23.0;26.5)	24.0 (22.3;29.5)
SF36 physical health						
1 month	55.9 (37.0;57.6)†	45.9 (37.7;55.5)	44.5 (32.4;53.1)†	43.6 (30.6;52.4)	44.9 (33.8;54.1)	53.6 (41.3;57.1)
6 months	51.8 (34.4;53.2)	45.5 (44.4;56.3)	43.3 (35.4;51.6)	49.8 (31.1;58.2)	49.0 (37.5;55.2)	51.1 (40.1;56.4)
12 months	51.5 (48.0;56.0)†	49.2 (40.2;57.8)†	39.0 (27.7;55.4)†	49.3 (33.6;56.3)	48.7 (37.9;54.4)	53.3 (49.7;56.2)
24 months	43.5 (38.7;52.3)†	52.5 (34.0;56.6)	36.0 (21.2;52.2)	43.9 (29.3;54.3)	50.0 (39.5;52.9)	49.6 (39.6;55.7)
SF36 mental health						
1 month	32.0 (29.8;47.3)†	46.9 (42.5;58.2)	49.9 (40.9;56.3)‡	55.3 (49.4;57.5)	52.6 (41.8;59.4)	50.4 (34.2;56.0)
6 months	40.7 (30.1;55.8)	46.2 (34.6;57.6)	54.7 (49.5;58.9)	52.4 (49.3;59.4)	55.9 (49.9;58.0)	52.8 (44.1;56.8)
12 months	37.8 (22.6;50.8)†	45.2 (36.6;50.7)	55.5 (40.3;58.8)	54.3 (52.4;56.5)	57.4 (49.4;60.5)	53.3 (44.2;56.0)
24 months	37.9 (24.5;51.9)†	49.8 (37.5;55.1)	52.1 (48.4;58.0)	54.2 (51.7;59.5)	53.4 (43.9;57.1)	51.5 (39.2;57.9)

Data are median (Q1;Q3). †p≤0.05 vs corresponding matched controls, ‡p≤0.05 vs anterior thalamic stroke patients, §p≤0.05 vs inferolateral thalamic stroke patients, #p≤0.05 vs paramedian thalamic stroke patients. HADS, Hospital Anxiety and Depression Scale; NAA, Nürnberger-Alters-Alltagsaktivitäten scale; SF36, Short Form-36

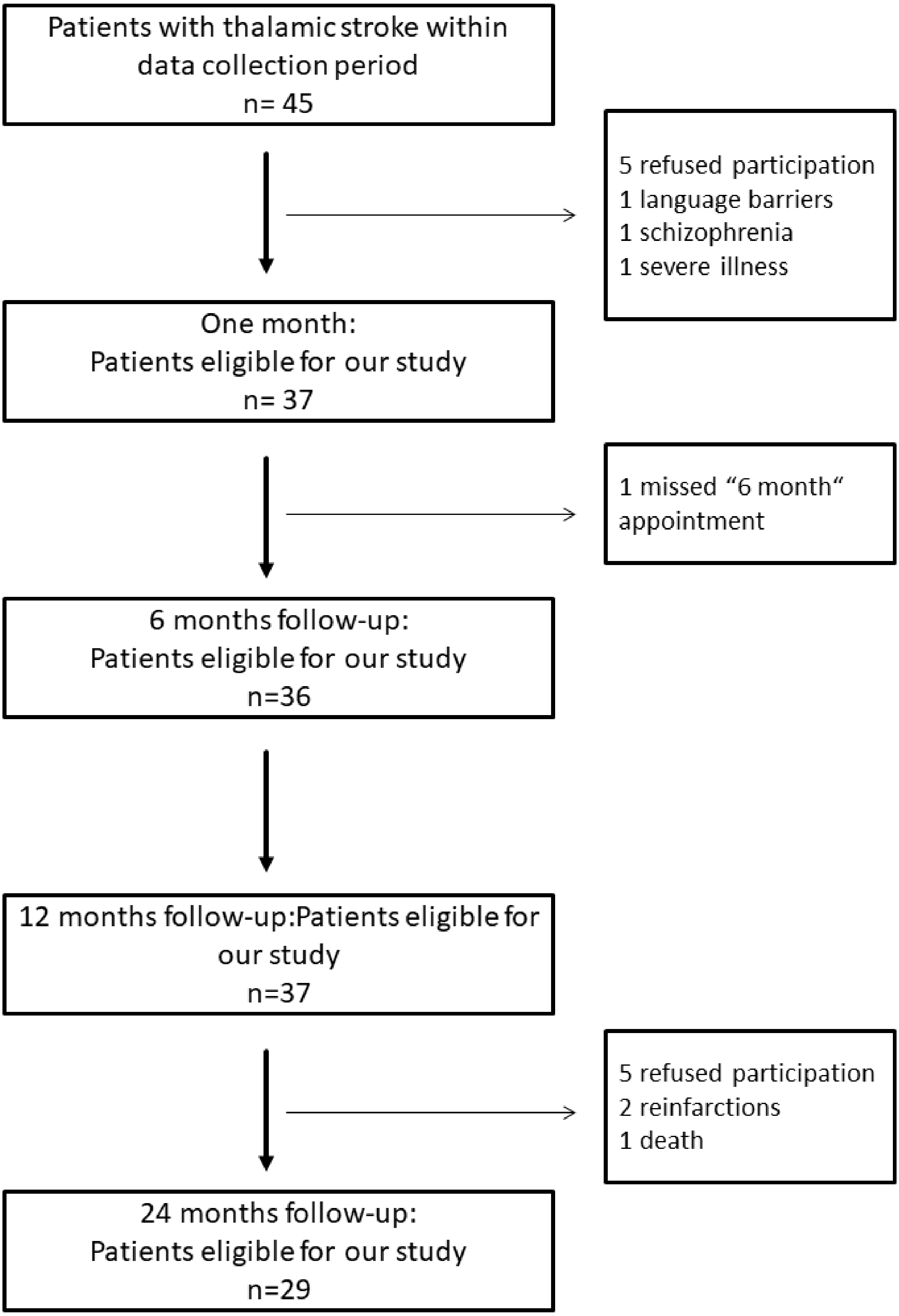

Figure S1. Thalamic stroke patient recruitment. Patients included and excluded at various points of examination are shown.
